# 1-(1-Decyl-2-oxoindolin-3-yl­idene)thio­semicarbazide

**DOI:** 10.1107/S1600536810016521

**Published:** 2010-05-15

**Authors:** Khalil Mamari, Abdelfettah Zerzouf, Hafid Zouihri, El Mokhtar Essassi, Seik Weng Ng

**Affiliations:** a Laboratoire de Chimie Organique Hétérocyclique, Pôle de Compétences Pharmacochimie, Université Mohammed V-Agdal, BP 1014 Avenue Ibn Batout, Rabat, Morocco; b Laboratoire de Chimie Organique et Etudes Physicochimiques, ENS Rabat, Morocco; c CNRST Division UATRS, Angle Allal Fassi/FAR, BP 8027 Hay Riad, Rabat, Morocco; d Department of Chemistry, University of Malaya, 50603 Kuala Lumpur, Malaysia

## Abstract

In the title 1-alkyl­isatin 3-thio­semicarbazone, C_19_H_28_N_4_OS, the imine C=N bond has a *Z* configuration, whereas the N—N—C=S unit has an *E* conformation. In the crystal, mol­ecules are connected by N—H⋯O hydrogen bonds, forming zigzag chains running along the *b* axis.

## Related literature

For background to *N*-substituted isatins and their derivatives, see: Bouhfid *et al.* (2008 ▶).
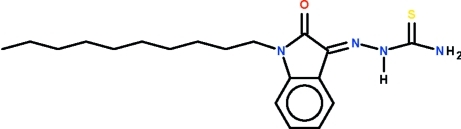


## Experimental

### 

#### Crystal data

C_19_H_28_N_4_OS
*M* *_r_* = 360.51
Monoclinic, 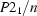

*a* = 11.8856 (2) Å
*b* = 11.0055 (2) Å
*c* = 15.1638 (3) Å
β = 99.468 (1)°
*V* = 1956.51 (6) Å^3^
*Z* = 4
Mo *K*α radiation
μ = 0.18 mm^−1^
*T* = 180 K
0.23 × 0.14 × 0.12 mm

#### Data collection

Bruker X8 APEXII diffractometer
Absorption correction: multi-scan (*SADABS*; Sheldrick, 1996 ▶) *T*_min_ = 0.960, *T*_max_ = 0.979
26304 measured reflections
5715 independent reflections
4227 reflections with *I* > 2σ(*I*)
*R*_int_ = 0.043

#### Refinement

*R*[*F* ^2^ > 2σ(*F* ^2^)] = 0.042
*wR*(*F* ^2^) = 0.135
*S* = 1.06
5715 reflections
238 parameters
3 restraints
H atoms treated by a mixture of independent and constrained refinement
Δρ_max_ = 0.30 e Å^−3^
Δρ_min_ = −0.36 e Å^−3^

### 

Data collection: *APEX2* (Bruker, 2008 ▶); cell refinement: *SAINT* (Bruker, 2008 ▶); data reduction: *SAINT*; program(s) used to solve structure: *SHELXS97* (Sheldrick, 2008 ▶); program(s) used to refine structure: *SHELXL97* (Sheldrick, 2008 ▶); molecular graphics: *X-SEED* (Barbour, 2001 ▶); software used to prepare material for publication: *publCIF* (Westrip, 2010 ▶).

## Supplementary Material

Crystal structure: contains datablocks global, I. DOI: 10.1107/S1600536810016521/bt5265sup1.cif

Structure factors: contains datablocks I. DOI: 10.1107/S1600536810016521/bt5265Isup2.hkl

Additional supplementary materials:  crystallographic information; 3D view; checkCIF report

## Figures and Tables

**Table 1:** Hydrogen-bond geometry (Å, °)

*D*—H⋯*A*	*D*—H	H⋯*A*	*D*⋯*A*	*D*—H⋯*A*
N1—H11⋯O1^i^	0.86 (1)	2.19 (1)	2.986 (2)	154 (2)

Symmetry code: (i) 
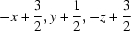
.

**Fig. 1.**
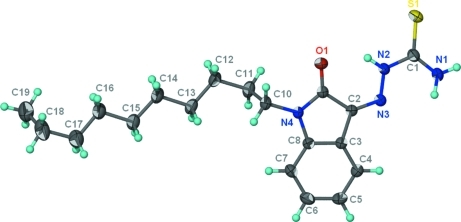
Anisotropic displacement ellipsoid plot (Barbour, 2001) of the molecule of C19H28N4OS at the 70% probability level; hydrogen atoms are drawn as spheres of arbitrary radius.

**Fig. 2.**
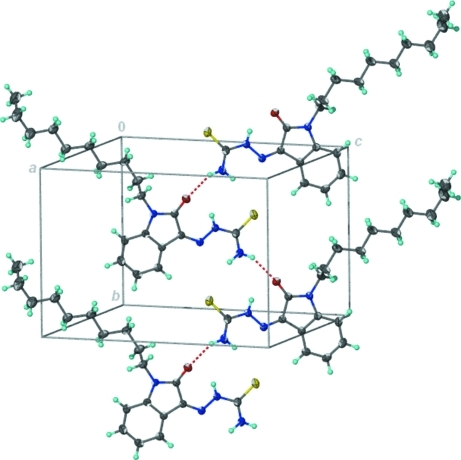
Packinig diagram of the title compound with hydrogen bonds drawn as dashed lines.

## supplementary crystallographic information

### Comment

*N*-Substituted isatins (Bouhfid *et al.*, 2008) represent a large family of heterocyclic compounds reported to show a wide range of useful medicinal properties. These condense readily with thiosemicarbazides to form crystalline thiosemicarbazones. The title 1-decylisatin derivative has a *Z* configuration around the imine C=N bond and *E* configuration the C(=S)–NH_2_ bond (Scheme I, Fig. 1). The molecules form zigzag chains though N–H···O hydrogen bonds along the *b*-axis of the monoclinic unit cell (Fig. 2). The decyl chain adopts an irregular zigzag conformation; the irregular nature lead to a compact packing and there are no voids in the crystal.

### Experimental

1-Decyl-isatin (1 g, 3.1 mmol) and thiosemicarbazide (0.31 g, 3.4 mmol) were dissolved in aqueous ethanol (50 ml); a few drops of glacial acetic acid were added. The mixture was heated for 4 hours. Yellow crystals separated from the cool solution in 80% yield.

### Refinement

Carbon-bound H-atoms were placed in calculated positions (C—H 0.95–0.99 Å) and were included in the refinement in the riding model approximation, with *U*(H) set to 1.2–1.5*U*_eq_(C). The amino-H atoms were located in a difference Fourier map, and they were refined isotropically with the N–H distance restrained to 0.86±0.01 Å.

### Crystal data

**Table:**

C_19_H_28_N_4_OS	*F*(000) = 776
*M**_r_* = 360.51	*D*_x_ = 1.224 Mg m^−^^3^
Monoclinic, *P*2_1_/*n*	Mo *K*α radiation, λ = 0.71073 Å
Hall symbol: -P 2yn	Cell parameters from 5658 reflections
*a* = 11.8856 (2) Å	θ = 2.3–28.7°
*b* = 11.0055 (2) Å	µ = 0.18 mm^−^^1^
*c* = 15.1638 (3) Å	*T* = 180 K
β = 99.468 (1)°	Prism, yellow
*V* = 1956.51 (6) Å^3^	0.23 × 0.14 × 0.12 mm
*Z* = 4	

### Data collection

**Table:**

Bruker X8 APEXII diffractometer	5715 independent reflections
Radiation source: fine-focus sealed tube	4227 reflections with *I* > 2σ(*I*)
graphite	*R*_int_ = 0.043
φ and ω scans	θ_max_ = 30.0°, θ_min_ = 2.0°
Absorption correction: multi-scan (*SADABS*; Sheldrick, 1996)	*h* = −16→16
*T*_min_ = 0.960, *T*_max_ = 0.979	*k* = −13→15
26304 measured reflections	*l* = −18→21

### Refinement

**Table:**

Refinement on *F*^2^	Primary atom site location: structure-invariant direct methods
Least-squares matrix: full	Secondary atom site location: difference Fourier map
*R*[*F*^2^ > 2σ(*F*^2^)] = 0.042	Hydrogen site location: inferred from neighbouring sites
*wR*(*F*^2^) = 0.135	H atoms treated by a mixture of independent and constrained refinement
*S* = 1.06	*w* = 1/[σ^2^(*F*_o_^2^) + (0.0721*P*)^2^ + 0.276*P*] where *P* = (*F*_o_^2^ + 2*F*_c_^2^)/3
5715 reflections	(Δ/σ)_max_ = 0.001
238 parameters	Δρ_max_ = 0.30 e Å^−^^3^
3 restraints	Δρ_min_ = −0.36 e Å^−^^3^

### Fractional atomic coordinates and isotropic or equivalent isotropic displacement parameters (Å^2^)

**Table:**

	*x*	*y*	*z*	*U*_iso_*/*U*_eq_	
S1	0.80459 (3)	0.78130 (4)	0.66953 (3)	0.03232 (12)	
O1	0.66844 (10)	0.67702 (9)	0.91795 (7)	0.0330 (3)	
N1	0.78312 (11)	1.00870 (12)	0.72565 (9)	0.0304 (3)	
N2	0.73941 (11)	0.85612 (11)	0.81634 (8)	0.0277 (3)	
N3	0.71786 (10)	0.93926 (10)	0.87721 (8)	0.0241 (2)	
N4	0.61721 (10)	0.76378 (10)	1.04405 (8)	0.0232 (2)	
C1	0.77480 (11)	0.89065 (12)	0.73872 (9)	0.0240 (3)	
C2	0.68081 (11)	0.89632 (12)	0.94641 (9)	0.0221 (3)	
C3	0.65089 (11)	0.96609 (12)	1.02047 (9)	0.0214 (3)	
C4	0.65109 (12)	1.08920 (12)	1.03890 (10)	0.0250 (3)	
H4	0.6761	1.1465	0.9994	0.030*	
C5	0.61370 (12)	1.12684 (13)	1.11687 (10)	0.0289 (3)	
H5	0.6136	1.2109	1.1310	0.035*	
C6	0.57670 (12)	1.04303 (14)	1.17397 (10)	0.0296 (3)	
H6	0.5518	1.0710	1.2268	0.035*	
C7	0.57503 (12)	0.91890 (13)	1.15612 (9)	0.0266 (3)	
H7	0.5491	0.8618	1.1953	0.032*	
C8	0.61282 (11)	0.88257 (11)	1.07871 (9)	0.0214 (3)	
C9	0.65662 (12)	0.76577 (12)	0.96510 (9)	0.0240 (3)	
C10	0.58378 (12)	0.65187 (12)	1.08470 (10)	0.0258 (3)	
H10A	0.5452	0.5974	1.0372	0.031*	
H10B	0.5286	0.6716	1.1249	0.031*	
C11	0.68480 (13)	0.58614 (14)	1.13747 (11)	0.0329 (3)	
H11A	0.7232	0.6404	1.1852	0.040*	
H11B	0.7402	0.5667	1.0974	0.040*	
C12	0.64970 (14)	0.46857 (13)	1.17964 (11)	0.0319 (3)	
H12A	0.5931	0.4257	1.1352	0.038*	
H12B	0.7175	0.4154	1.1935	0.038*	
C13	0.59920 (13)	0.48675 (12)	1.26434 (11)	0.0305 (3)	
H13A	0.5286	0.5355	1.2500	0.037*	
H13B	0.6539	0.5334	1.3079	0.037*	
C14	0.57148 (13)	0.36767 (13)	1.30730 (11)	0.0314 (3)	
H14A	0.6407	0.3161	1.3168	0.038*	
H14B	0.5121	0.3243	1.2655	0.038*	
C15	0.52981 (13)	0.38398 (14)	1.39615 (11)	0.0321 (3)	
H15A	0.5876	0.4308	1.4369	0.039*	
H15B	0.4588	0.4326	1.3860	0.039*	
C16	0.50680 (14)	0.26574 (14)	1.44180 (11)	0.0341 (3)	
H16A	0.5792	0.2204	1.4577	0.041*	
H16B	0.4540	0.2153	1.3996	0.041*	
C17	0.45517 (16)	0.28689 (16)	1.52591 (13)	0.0431 (4)	
H17A	0.3889	0.3420	1.5111	0.052*	
H17B	0.5123	0.3286	1.5706	0.052*	
C18	0.41613 (17)	0.17156 (19)	1.56828 (14)	0.0511 (5)	
H18A	0.3704	0.1947	1.6146	0.061*	
H18B	0.3663	0.1242	1.5219	0.061*	
C19	0.5135 (2)	0.09282 (19)	1.60994 (14)	0.0567 (5)	
H19A	0.4836	0.0208	1.6363	0.085*	
H19B	0.5625	0.1386	1.6567	0.085*	
H19C	0.5579	0.0676	1.5641	0.085*	
H11	0.8010 (16)	1.0349 (19)	0.6760 (9)	0.048 (6)*	
H12	0.7606 (16)	1.0593 (15)	0.7628 (11)	0.044 (5)*	
H2	0.7284 (16)	0.7811 (10)	0.8277 (13)	0.044 (5)*	

### Atomic displacement parameters (Å^2^)

**Table:**

	*U*^11^	*U*^22^	*U*^33^	*U*^12^	*U*^13^	*U*^23^
S1	0.0421 (2)	0.0309 (2)	0.0277 (2)	0.00066 (15)	0.01700 (16)	−0.00417 (15)
O1	0.0523 (7)	0.0207 (5)	0.0300 (6)	0.0001 (4)	0.0186 (5)	−0.0034 (4)
N1	0.0425 (7)	0.0262 (6)	0.0262 (6)	0.0024 (5)	0.0164 (6)	0.0043 (5)
N2	0.0427 (7)	0.0197 (5)	0.0247 (6)	0.0004 (5)	0.0175 (5)	0.0007 (5)
N3	0.0297 (6)	0.0216 (5)	0.0227 (6)	0.0011 (4)	0.0095 (5)	0.0007 (4)
N4	0.0316 (6)	0.0180 (5)	0.0222 (6)	0.0008 (4)	0.0106 (5)	0.0025 (4)
C1	0.0247 (6)	0.0266 (6)	0.0216 (6)	0.0011 (5)	0.0069 (5)	0.0015 (5)
C2	0.0268 (6)	0.0193 (6)	0.0214 (6)	0.0013 (5)	0.0074 (5)	0.0007 (5)
C3	0.0242 (6)	0.0199 (6)	0.0207 (6)	0.0006 (5)	0.0052 (5)	0.0003 (5)
C4	0.0287 (7)	0.0208 (6)	0.0262 (7)	−0.0004 (5)	0.0062 (5)	0.0009 (5)
C5	0.0350 (7)	0.0226 (6)	0.0293 (8)	0.0010 (5)	0.0064 (6)	−0.0063 (5)
C6	0.0346 (8)	0.0320 (7)	0.0233 (7)	0.0021 (6)	0.0081 (6)	−0.0056 (6)
C7	0.0323 (7)	0.0281 (7)	0.0209 (7)	0.0007 (5)	0.0087 (5)	0.0007 (5)
C8	0.0246 (6)	0.0198 (6)	0.0202 (6)	0.0020 (5)	0.0048 (5)	0.0005 (5)
C9	0.0306 (7)	0.0204 (6)	0.0226 (6)	0.0006 (5)	0.0091 (5)	0.0015 (5)
C10	0.0313 (7)	0.0202 (6)	0.0274 (7)	−0.0029 (5)	0.0095 (6)	0.0035 (5)
C11	0.0336 (7)	0.0268 (7)	0.0400 (9)	−0.0001 (6)	0.0107 (6)	0.0105 (6)
C12	0.0383 (8)	0.0219 (6)	0.0370 (8)	0.0034 (6)	0.0104 (7)	0.0079 (6)
C13	0.0341 (7)	0.0207 (6)	0.0373 (8)	−0.0011 (5)	0.0077 (6)	0.0058 (6)
C14	0.0350 (8)	0.0240 (7)	0.0361 (8)	0.0001 (6)	0.0082 (6)	0.0077 (6)
C15	0.0345 (8)	0.0281 (7)	0.0345 (8)	−0.0001 (6)	0.0076 (6)	0.0064 (6)
C16	0.0367 (8)	0.0304 (7)	0.0366 (9)	0.0013 (6)	0.0102 (7)	0.0080 (6)
C17	0.0451 (9)	0.0427 (10)	0.0453 (10)	0.0034 (7)	0.0186 (8)	0.0095 (8)
C18	0.0539 (11)	0.0584 (12)	0.0445 (11)	−0.0115 (9)	0.0185 (9)	0.0095 (9)
C19	0.0836 (15)	0.0492 (11)	0.0392 (10)	−0.0012 (10)	0.0159 (10)	0.0104 (9)

### Geometric parameters (Å, °)

**Table:**

S1—C1	1.6723 (14)	C11—C12	1.531 (2)
O1—C9	1.2320 (17)	C11—H11A	0.9900
N1—C1	1.3204 (18)	C11—H11B	0.9900
N1—H11	0.864 (9)	C12—C13	1.518 (2)
N1—H12	0.865 (9)	C12—H12A	0.9900
N2—N3	1.3537 (16)	C12—H12B	0.9900
N2—C1	1.3673 (17)	C13—C14	1.5235 (19)
N2—H2	0.858 (9)	C13—H13A	0.9900
N3—C2	1.2924 (17)	C13—H13B	0.9900
N4—C9	1.3556 (17)	C14—C15	1.521 (2)
N4—C8	1.4132 (16)	C14—H14A	0.9900
N4—C10	1.4613 (17)	C14—H14B	0.9900
C2—C3	1.4521 (18)	C15—C16	1.520 (2)
C2—C9	1.5014 (18)	C15—H15A	0.9900
C3—C4	1.3833 (18)	C15—H15B	0.9900
C3—C8	1.4004 (18)	C16—C17	1.521 (2)
C4—C5	1.3928 (19)	C16—H16A	0.9900
C4—H4	0.9500	C16—H16B	0.9900
C5—C6	1.385 (2)	C17—C18	1.529 (3)
C5—H5	0.9500	C17—H17A	0.9900
C6—C7	1.392 (2)	C17—H17B	0.9900
C6—H6	0.9500	C18—C19	1.499 (3)
C7—C8	1.3831 (18)	C18—H18A	0.9900
C7—H7	0.9500	C18—H18B	0.9900
C10—C11	1.513 (2)	C19—H19A	0.9800
C10—H10A	0.9900	C19—H19B	0.9800
C10—H10B	0.9900	C19—H19C	0.9800
			
C1—N1—H11	119.6 (15)	C13—C12—C11	114.51 (13)
C1—N1—H12	119.8 (13)	C13—C12—H12A	108.6
H11—N1—H12	119.9 (19)	C11—C12—H12A	108.6
N3—N2—C1	121.24 (12)	C13—C12—H12B	108.6
N3—N2—H2	117.5 (13)	C11—C12—H12B	108.6
C1—N2—H2	121.3 (13)	H12A—C12—H12B	107.6
C2—N3—N2	115.75 (11)	C12—C13—C14	113.07 (12)
C9—N4—C8	110.65 (11)	C12—C13—H13A	109.0
C9—N4—C10	122.93 (11)	C14—C13—H13A	109.0
C8—N4—C10	126.41 (11)	C12—C13—H13B	109.0
N1—C1—N2	116.36 (12)	C14—C13—H13B	109.0
N1—C1—S1	125.81 (11)	H13A—C13—H13B	107.8
N2—C1—S1	117.83 (10)	C13—C14—C15	113.72 (13)
N3—C2—C3	126.45 (12)	C13—C14—H14A	108.8
N3—C2—C9	127.20 (12)	C15—C14—H14A	108.8
C3—C2—C9	106.33 (11)	C13—C14—H14B	108.8
C4—C3—C8	120.36 (12)	C15—C14—H14B	108.8
C4—C3—C2	132.97 (12)	H14A—C14—H14B	107.7
C8—C3—C2	106.64 (11)	C16—C15—C14	114.32 (13)
C3—C4—C5	118.21 (13)	C16—C15—H15A	108.7
C3—C4—H4	120.9	C14—C15—H15A	108.7
C5—C4—H4	120.9	C16—C15—H15B	108.7
C6—C5—C4	120.72 (13)	C14—C15—H15B	108.7
C6—C5—H5	119.6	H15A—C15—H15B	107.6
C4—C5—H5	119.6	C15—C16—C17	112.24 (14)
C5—C6—C7	121.85 (13)	C15—C16—H16A	109.2
C5—C6—H6	119.1	C17—C16—H16A	109.2
C7—C6—H6	119.1	C15—C16—H16B	109.2
C8—C7—C6	116.91 (13)	C17—C16—H16B	109.2
C8—C7—H7	121.5	H16A—C16—H16B	107.9
C6—C7—H7	121.5	C16—C17—C18	114.67 (16)
C7—C8—C3	121.95 (12)	C16—C17—H17A	108.6
C7—C8—N4	128.32 (12)	C18—C17—H17A	108.6
C3—C8—N4	109.70 (11)	C16—C17—H17B	108.6
O1—C9—N4	126.06 (12)	C18—C17—H17B	108.6
O1—C9—C2	127.26 (12)	H17A—C17—H17B	107.6
N4—C9—C2	106.67 (11)	C19—C18—C17	112.98 (16)
N4—C10—C11	112.26 (12)	C19—C18—H18A	109.0
N4—C10—H10A	109.2	C17—C18—H18A	109.0
C11—C10—H10A	109.2	C19—C18—H18B	109.0
N4—C10—H10B	109.2	C17—C18—H18B	109.0
C11—C10—H10B	109.2	H18A—C18—H18B	107.8
H10A—C10—H10B	107.9	C18—C19—H19A	109.5
C10—C11—C12	112.20 (12)	C18—C19—H19B	109.5
C10—C11—H11A	109.2	H19A—C19—H19B	109.5
C12—C11—H11A	109.2	C18—C19—H19C	109.5
C10—C11—H11B	109.2	H19A—C19—H19C	109.5
C12—C11—H11B	109.2	H19B—C19—H19C	109.5
H11A—C11—H11B	107.9		
			
C1—N2—N3—C2	177.49 (13)	C10—N4—C8—C7	−1.2 (2)
N3—N2—C1—N1	−2.0 (2)	C9—N4—C8—C3	−0.06 (16)
N3—N2—C1—S1	177.49 (10)	C10—N4—C8—C3	−179.47 (12)
N2—N3—C2—C3	−179.58 (13)	C8—N4—C9—O1	−178.82 (14)
N2—N3—C2—C9	−1.7 (2)	C10—N4—C9—O1	0.6 (2)
N3—C2—C3—C4	0.7 (2)	C8—N4—C9—C2	0.34 (15)
C9—C2—C3—C4	−177.54 (15)	C10—N4—C9—C2	179.77 (12)
N3—C2—C3—C8	178.72 (13)	N3—C2—C9—O1	0.4 (2)
C9—C2—C3—C8	0.44 (14)	C3—C2—C9—O1	178.66 (14)
C8—C3—C4—C5	0.6 (2)	N3—C2—C9—N4	−178.74 (13)
C2—C3—C4—C5	178.39 (14)	C3—C2—C9—N4	−0.48 (15)
C3—C4—C5—C6	−0.4 (2)	C9—N4—C10—C11	83.30 (17)
C4—C5—C6—C7	−0.1 (2)	C8—N4—C10—C11	−97.36 (16)
C5—C6—C7—C8	0.4 (2)	N4—C10—C11—C12	−179.72 (12)
C6—C7—C8—C3	−0.2 (2)	C10—C11—C12—C13	−78.90 (18)
C6—C7—C8—N4	−178.24 (14)	C11—C12—C13—C14	−176.76 (13)
C4—C3—C8—C7	−0.4 (2)	C12—C13—C14—C15	175.33 (13)
C2—C3—C8—C7	−178.65 (12)	C13—C14—C15—C16	−177.44 (13)
C4—C3—C8—N4	178.04 (12)	C14—C15—C16—C17	−174.78 (14)
C2—C3—C8—N4	−0.25 (15)	C15—C16—C17—C18	172.62 (15)
C9—N4—C8—C7	178.21 (14)	C16—C17—C18—C19	69.9 (2)

### Hydrogen-bond geometry (Å, °)

**Table:**

*D*—H···*A*	*D*—H	H···*A*	*D*···*A*	*D*—H···*A*
N1—H12···S1^i^	0.87 (1)	2.81 (1)	3.628 (1)	159 (2)
N1—H11···O1^i^	0.86 (1)	2.19 (1)	2.986 (2)	154 (2)

Symmetry codes: (i) −*x*+3/2, *y*+1/2, −*z*+3/2.
